# Supplementary material for: Willingness to participate in future HIV vaccine trials among men who have sex with men and female sex workers living in Nairobi, Kenya
Source: PLoS One. 2020 Aug 24;15(8):e0238028. doi: 10.1371/journal.pone.0238028 (PMC7444816; doi:10.1371/journal.pone.0238028)
Supplement: S1 File — (PDF) [file pone.0238028.s001.pdf]

RISK ASSESSMENT QUESTIONNAIRE

I VOLUNTEER INFORMATION

Study ID: SiVET- ¦___¦___¦-|___¦___¦___¦___¦ **Visit Number**: ¦__¦__¦ ¦__¦
(alpha)

II VISIT INFORMATION

Date of assessment: ¦__¦__¦¦__¦__¦__¦¦__¦__¦__¦__¦
(ddMMMyyyy)

1. During the last month on average, how often have you had a drink containing alcohol?
   ☐ None ☐ Daily ☐ Weekly ☐ 1-3 Times/month

If the answer is "None", skip to question 4.

2. During the last month, how often were you drunk/drinking alcohol before sex?

☐ Never ☐ Sometimes (less than half the time) ☐ Frequently (more than half the time) ☐ Always

☐ Not sexually active

3. **CAGE SCORE:** *(Scoring "YES" = 1 "NO"= 0. Total score is the sum of "a" to "d". A score of ≥2 indicates an alcohol problem.* ***AT ENROLMENT: Conduct full alcohol addiction assessment as per site SOP for scores ≥2****)*

a.	Have you ever felt you should cut down on your drinking?	Yes ☐	No ☐
b.	Have people annoyed you by criticizing your drinking?	Yes ☐	No ☐
c.	Have you ever felt bad or guilty about your drinking?	Yes ☐	No ☐
d.	Have you ever had a drink first thing in the morning (as an eye opener) to steady your nerves or get rid of a hangover?	Yes ☐	No ☐
e.	Total Score______		

4. In the last month, which of the following, if any, have you used?	**No**	**Yes**	**Don't Know**
Injecting illegal or recreational drugs with a needle	☐	☐	☐
Marijuana	☐	☐	☐
Crack/cocaine	☐	☐	☐
Other, specify: (*30 character limit*) ______________________	☐	☐	☐
Other, specify: (*30 character limit*) ______________________	☐	☐	☐
Other, specify: (*30 character limit*) ______________________	☐	☐	☐

NOTE: Speak this aloud to the volunteer:
Now we would like to ask you about your sexual partners and condom use. Some of these questions may be sensitive or uncomfortable for you, but everyone is asked the same questions. Some questions may not be relevant to you. Remember that all answers you give are confidential. It is very important for this research study that you give honest answers. When we talk about sex, we mean when you put your penis into a partner's vagina or anus, or a partner puts his penis into your vagina or anus.

5. In the last month, how many sex partners did you have?

¦__¦__¦ ☐ Too many to remember

***If answer is "00" proceed to Question 10*.**

5a. In the last month, how frequently did you use condoms when having sex?

☐ Never ☐ Sometimes (less than half the time) ☐ Frequently (more than half the time) ☐ Always

5b. How many <u>new</u> partners did you have, that is, someone you never had sex with before?

¦__¦__¦ ☐ Too many to remember

<u>Men Only:</u>

6. In the last month, how many of your sex partners were male?

¦__¦__¦ ☐ Too many to remember

<u>Men and Women:</u>

7. Are any of these sex partners HIV infected?
☐ No ☐ Yes ☐ Don't Know

8. In the last month, have you had receptive (bottom) anal sex, that is, where your sex partner put his penis in your anus?

☐ No ☐ Yes ☐ Don't know ☐ Not Applicable (if answer to Q6=00)

For Men only

9. In the last month, have you had insertive (top) anal sex, that is, put your penis in your sex partner's anus?

☐ No ☐ Yes ☐ Don't know

HIV PREVENTION MODALITIES *(Do not prompt volunteers with any answers)*

10. How do you think the spread of HIV/AIDS can be prevented from an infected person to an uninfected person **(check all that apply)**?

|__| Abstain from sex

|__| Use a condom when having sex

|__| Limit sex to one partner (monogamy)

|__| Limit number of sex partners

|__| Taking HIV medicines BEFORE un-protected sex (Pre-exposure prophylaxis)

|__| Taking HIV medicines AFTER un-protected sex (Post-exposure prophylaxis)

|__| Voluntary Medical Male Circumcision

|__| Make sure all injections are done with clean needles

|__| Avoid sharing razors / blades/ injection needles

|__| PMTCT / HIV medications for HIV + pregnant mothers

|__| Provide HIV medicines for HIV + people (Treatment as Prevention)

|__| Unsure or no answer

|__| Other, specify: ________________________________
____

11. What HIV prevention methods have you used in the last 6months **(check all that apply)**?

|__| Abstain from sex

|__| Use a condom when having sex

|__| Limit sex to one partner (monogamy)

|__| Limit number of sex partners

|__| Taking HIV medicines BEFORE un-protected sex (Pre-exposure prophylaxis)

|__| Taking HIV medicines AFTER un-protected sex (Post-exposure prophylaxis)

|__| Make sure all injections are done with clean needles

|__| Avoid sharing razors / blades/ injection needles

|__| HIV + partner on treatment (Treatment as Prevention)

|__| Voluntary Medical Male Circumcision

|__| None

|__| Other, specify: ________________________________
____

Form Completed by	____________ Signature	____________ Printed Name/Initials	____________ Date Form Signed
Reviewed by	____________ Signature	____________ Printed Name/Initials	____________ Date Form Signed
Entered by	____________ Signature	____________ Printed Name/Initials	____________ Date Form Signed
