# Supplementary material for: Willingness to participate in future HIV vaccine trials among men who have sex with men and female sex workers living in Nairobi, Kenya
Source: PLoS One. 2020 Aug 24;15(8):e0238028. doi: 10.1371/journal.pone.0238028 (PMC7444816; doi:10.1371/journal.pone.0238028)
Supplement: S2 File — (PDF) [file pone.0238028.s002.pdf]

SiVET STUDY

Vaccine Trial Willingness Questionnaire

To be completed by Community Mobilizer

I VOLUNTEER INFORMATION

Study ID: SiVET- ¦___¦___¦-¦___¦___¦___¦___¦ **Visit Number**: ¦__¦__¦ ¦__¦
(alpha)

II VISIT INFORMATION

Date of assessment: ¦__¦__¦¦__¦__¦__¦¦__¦__¦__¦__¦
(ddMMMyyyy)

1. How would you describe your overall experience in this study ***(read choices to volunteer, volunteer chooses only one)***?

☐ Very Good ☐ Good ☐ Neither good nor bad ☐ Bad ☐ Very Bad

2. Explain what you liked about your experience with this study ***(Do not prompt. Tick all that apply)***:

|__| I had no good experience in this study
|__| Compensation for travel
|__| Receiving education about HIV
|__| Altruism (Feel good to help)
|__| Health Care
|__| Health Education
|__| Staff were helpful/nice
|__| Regular HIV VCT
|__| Knowing I am protected against Hepatitis B
|__| Information about HIV risk reduction
|__| Other Explain: ________________________________
____

3. Explain what you DID NOT like about your experience with this study ***(Do not Prompt. Tick all that apply)***

|__| I had no bad experience in this study
|__| Having my blood drawn
|__| Contraception/birth control requirements
|__| Shame/fear that others would be judgmental in a negative way towards you
|__| Fear that participation might be disclosed to others
|__| Fear that HIV status might be disclosed to others
|__| Time commitment
|__| Not enough compensation for travel/time
|__| Staff were judgemental
|__| Other, explain: ________________________________
____

4. Would you be willing to participate in a HIV-Vaccine trial if it had exactly the same procedures as the study you have just completed ***(read choices to volunteer, volunteer chooses only one)****?*

|__| Yes (Very likely)
|__| Maybe yes (Somewhat likely)
|__| Don't know
|__| Maybe Not (Not likely)
|__| No (Not at all)

4a. If volunteer answered “Yes” or “Maybe yes” Explain why ***(Do not prompt. Tick all that apply)***
|__| Altruism (feels good to help)
|__| To get the education about HIV
|__| To get the health care
|__| To get the regular HIV VCT
|__| Hope of being protected against getting HIV
|__| Other, explain:____

4b. If volunteer answered “No” or “Maybe Not” Explain why ***(Do not Prompt. Tick all that apply)***
|__| Anxiety/fear of catching HIV from vaccine
|__| Concerns over blood draw
|__| Contraception/birth control requirements
|__| Shame/fear that others would be judgmental in a negative way towards you
|__| Time commitment
|__| Compensation for time/travel not worth it
|__| Fear that participation might be disclosed to others
|__| Fear that HIV status might be disclosed to others
|__| Other, explain: ____

5. Would you be willing to participate in an HIV vaccine trial that is longer than 2 years ***(read choices to volunteer, volunteer chooses only one)***?
|__| Yes (Very likely)
|__| Maybe yes (Somewhat likely)
|__| Don’t know
|__| Maybe Not (Not likely)
|__| No (Not at all)

6. Would you be willing to provide more blood than you did for this study for an HIV vaccine study ***(read choices to volunteer, volunteer chooses only one)***?
|__| Yes (Very likely)
|__| Maybe yes (Somewhat likely)
|__| Don’t know
|__| Maybe Not (Not likely)
|__| No (Not at all)

Form Completed by	____________________ Signature	____________________ Printed Name/Initials	________________ Date Form Signed
Reviewed by	____________________ Signature	____________________ Printed Name/Initials	________________ Date Form Signed
Entered by	____________________ Signature	____________________ Printed Name/Initials	________________ Date Form Signed
