# Supplementary material for: Willingness to participate in future HIV vaccine trials among men who have sex with men and female sex workers living in Nairobi, Kenya
Source: PLoS One. 2020 Aug 24;15(8):e0238028. doi: 10.1371/journal.pone.0238028 (PMC7444816; doi:10.1371/journal.pone.0238028)
Supplement: Attachment — Submitted filename: Response to reviewers.docx [file pone.0238028.s003.docx]

19-Feb-2020

Re: PONE-D-19-31011

**Willingness to participate in future HIV vaccine trials among men who have sex with men and female sex workers living in Nairobi, Kenya.**

Dear Dr. Newman,

Thank you for your comments on our manuscript.  Below, we list each comment, with our responses shown.  We look forward to hearing from you soon.

Sincerely,

Elizabeth Mutisya

1. Please ensure that your manuscript meets PLOS ONE's style requirements, including those for file naming. The PLOS ONE style templates can be found at
[http://www.journals.plos.org/plosone/s/file?id=wjVg/PLOSOne_formatting_sample_main_body.pdf](https://clicktime.symantec.com/3PcAFF38EkVumVQfWKMriq36H2?u=http%3A%2F%2Fwww.journals.plos.org%2Fplosone%2Fs%2Ffile%3Fid%3DwjVg%2FPLOSOne_formatting_sample_main_body.pdf) and [http://www.journals.plos.org/plosone/s/file?id=ba62/PLOSOne_formatting_sample_title_authors_affiliations.pdf](https://clicktime.symantec.com/3XeCEoraVgeBFKjy5Zqhi2N6H2?u=http%3A%2F%2Fwww.journals.plos.org%2Fplosone%2Fs%2Ffile%3Fid%3Dba62%2FPLOSOne_formatting_sample_title_authors_affiliations.pdf)

***Authors’ response: We have reviewed the above and confirm we meet these requirements***

2. Please include additional information regarding the survey or questionnaire used in the study and ensure that you have provided sufficient details that others could replicate the analyses. For instance, if you developed a questionnaire as part of this study and it is not under a copyright more restrictive than CC-BY, please include a copy, in both the original language and English, as Supporting Information.

***Authors’ response: Thank you for this comment; we have now included a copy of our questionnaire uploaded as supporting information. (S1 File PDF Risk assessment questionnaire)***

3. We note that you have stated that you will provide repository information for your data at acceptance. Should your manuscript be accepted for publication, we will hold it until you provide the relevant accession numbers or DOIs necessary to access your data. If you wish to make changes to your Data Availability statement, please describe these changes in your cover letter and we will update your Data Availability statement to reflect the information you provide.

***Authors’ response:*** ***The KAVI-ICR agrees to open data access. The data collected by KAVI-ICR can be made available to other bona fide researchers, upon application to access the necessary data from which this manuscript was generated. The corresponding and other co-author emails have been provided and they could be contacted anytime for further clarifications and/or support to access the data.***


Reviewer #1: Reviewer’s comments
The presented data is timely in the efforts to recruit key populations in HIV vaccine trials. Targeting men who have sex with men and female sex workers for future trials is essential because these are the most affected groups in SSA. Nevertheless, some parts of the manuscript need some revisions to increase clarity. These are:
Abstract
The conclusion lines 44-47 needs revision. This statement does not match the key results. The authors should modify this statement to increase clarity.

***Authors’ response: We have changed the language some to better reflect our findings in the results.***

Introduction
The first paragraph, from line 54… should start with the current global, regional, and national HIV prevalence, followed by specific key populations' HIV prevalence. Part of this description appears between lines 60 and 66.

***Authors’ response: Thank you for your comment. While we appreciate that it often seems like most HIV clinical research papers start this way, the important take home message here is that we are not on target to achieve upcoming milestones to reduce incidence, and that an HIV vaccine remains an important goal. To do this, we need volunteers for upcoming efficacy trials. We feel our research supports the inclusion of these key populations in future trials.***

Discussion
The authors should discuss their results before relating to others' work. See, for example, lines 215 – 216; what does the willingness of four of five respondents mean in the context of this study?

***Authors’ response: We have added another sentence to the beginning of the discussion text prior to discussing others’ work.***

Line 257-261, access to health services as a motivator to take part in HIV vaccine trials have been reported from other low-income countries besides Uganda and Kenya. It would be nice to expand this particular discussion by using additional related citations.

additionally, the authors must discuss the limitations and mitigations of this study. For example, the questionnaire was administered 12-15 months after the trial. Would this gap in terms of timing influence the obtained results?

***Authors’ response: Thank you for these comments. We have included references outside of Uganda and Kenya. We also kindly refer the reviewer to the last paragraph of the discussion, where we summarize limitations and mitigations. We also respectfully remind the reviewer that the questionnaire was administered 12-15 months after enrollment, on the last study visit.***

Conclusion
Lines 275 – 281, should be rephrased to match the current key study results. For example, the issue of improving recruitment and retention was not part of the key results. Although it is an important recommendation, I think other issues about the current results should be emphasized first.

***Authors’ response: Thank you for these comments. In this paragraph, we aren’t talking about improving recruitment and retention, instead we are noting that those who reported a better experience in the SiVET were more likely to report being willing to participate, suggesting that participants who felt their needs met were probably more likely to enroll into a future trial. Perhaps I am not understanding your question?***

Reviewer #2: The manuscript focuses on assessing the willingness to participate in future vaccine trials among MSM and female sex workers in Kenya. As the study assessed the willingness among the participants of a SiVET, it is claimed that the reported willingness maybe closer to the willingness to participate in actual trials.

Major comments:
1. The first sentence in the Introduction (line numbers 54, 55) seems to make a sweeping statement about achievement of success in care, treatment and prevention. This statement needs to be qualified and specified – i.e., which achievements are being referred to – biomedical or public health achievements?

***Authors’ response:***

***We have stated in the introduction section that “there are various approaches available to public health including behavioural, structural and biomedical intervention”***

2. It is not clear whether and what percentage of MSM participants engaged in sex work. That information may be important for future trials.

***Authors’ response: It would have been valuable information, however in this particular study we were not required to collect data regarding sex work.***

3. Given the findings that those with (good) knowledge about HIV biomedical prevention methods had lower odds to participate in future trials, it is important to know what were these prevention methods that were asked in the questionnaire (e.g., PrEP, microbicides?) Whether this information (on prevention methods) was provided as part of SiVET?

***Authors’ response: Both biomedical and behavioural HIV prevention methods were included in the questionnaire. We have also included the specific HIV preventive methods have also been in the text.***

***We have included the risk assessment questionnaire as an attachment. The information on prevention methods provided is found on page 3numbers 10 and 11. (S1 File PDF Risk assessment questionnaire)***

4. Sample size calculation: How the decision to enroll 200 MSM and 50 FSW was arrived at from the calculated sample size of 250?

***Authors’ response: Our decision to enroll 250 volunteers, 20% of whom would be FSW by design, was also based on feasibility considerations.  We were most interested in reaching out to the MSM communities in Nairobi where HIV risk remains high, however our partner SWOP works with both male and female sex workers.  To avoid stigma and bad feelings with our partners, we agreed to enroll a certain percentage of women into the study and settled on 20%.***

5. It is not clear what is meant by “…included in a backward elimination algorithm of multivariable logistic regression to arrive at a final model, retaining those variables with a p <0.05”. Whether it means only those variables with p <0.05 were included in the final multivariable model? But it does not seem to be the case (Table 2).

***Authors’ response: The decision to keep a variable in the multivariable model was dependent on the model log likelihood ratio test p-value for the inclusion of a given variable and not Wald p-value, except for age and trial population as these were considered a priori confounders.***

***We have corrected the text in our methods to make it clearer “Univariate logistic regression was used to evaluate the association of all variables with willingness to participate; factors associated with willingness to participate at p<0.20 on log likelihood test were included in the initial multivariable model (except for age and trial population that were included a priori). Factors were retained in the multivariable model if the log likelihood test p-value of inclusion of a factor was less than 0.05”.***

6. Whether the five new infections occurred among MSM or FSWs?

***Authors’ response: The five new HIV infections occurred among MSM.***

***We have added the information text in the results section and Fig 1 HIV infected. “5M: 0F”***

7. In the discussion section, the authors could comment on the reported reasons for not willing to participate: “time commitment, fear that their HIV status would be disclosed to others without their permission and the strict contraception requirements”. For example, the prevalence of HIV-related stigma/discrimination in Kenya seems to be associated with the second concern.

***Authors’ response: Information on the reported reasons for not willing to participate has been added in the discussion section.*** ***Additional related citations have been included in the reference.***

***We have expanded the discussion citing Newman PA, Duan N, Roberts KJ, Seiden D, Rudy ET, Swendeman D, et al. HIV vaccine trial participation among ethnic minority communities: barriers, motivators, and implications for recruitment. J Acquir Immune D***

***To comment on the “fear that their HIV status would be disclosed to others without their permission“ indicates stigma/discrimination and we have expanded in the discussion section that “although HIV/AIDS awareness is high in Kenya, key population groups still face HIV stigma, discrimination and violence hence, this may deter people from participation because it is related to HIV/AIDS“***

Minor comment:
There are minor language errors (e.g., line 146: “participants’ were classified”, which can be corrected by careful proofreading.

***Authors’ response: We have reviewed the paper again, and there are minor corrections and clarifications throughout that we hope make it clearer.***
