# Supplementary material for: Willingness to participate in future HIV vaccine trials among men who have sex with men and female sex workers living in Nairobi, Kenya
Source: PLoS One. 2020 Aug 24;15(8):e0238028. doi: 10.1371/journal.pone.0238028 (PMC7444816; doi:10.1371/journal.pone.0238028)
Supplement: Attachment — Submitted filename: Response to reviewers.docx [file pone.0238028.s004.docx]

30^th^ Jul 2020

RE: PONE-D-19-31011R1

**Willingness to participate in future HIV vaccine trials among men who have sex with men and female sex workers living in Nairobi, Kenya.**

Dear Dr. Newman,
Thank you for your comments on our manuscript. Below, we list each comment with our responses shown. We look forward to hearing from you soon.

Sincerely,

Elizabeth Mutisya

The authors have made a few of the revisions suggested by the two reviewers, and these are helpful. However, several of the reviewers' comments are not adequately responded to; and some are only responded to in the Reviewer's comments, with no corresponding changes in the manuscript. This is not sufficient. Also, please indicate the exact Line number in the manuscript where each revision has been made to ensure the reviewer/editor can locate them. This is more important since a few of the comments seem not to be responded to.

***Authors’ response: Thank you for the helpful suggestions. We have reviewed the manuscript and confirm corresponding changes to the document. We have indicated the exact line number where revision has been made.***

For example, Reviewer 1: "The first paragraph...should start with current global, regional and national prevalence." The response to the reviewer is inadequate.

***Authors’ response: Thank you for the clarification. We have included the current global, regional and national HIV prevalence and specific key population HIV prevalence in the first paragraph of the introduction and expanded the citation. Lines 51-70***

Abstract
The conclusion lines 44-47 needs revision. This statement does not match the key results. The authors should modify this statement to increase clarity 43-46

***Authors’ response: Thank you for the comment. We have changed the language to better emphasize our study findings lines 44-47***

In the conclusion: Your response, "Perhaps I am not understanding your question" is unfortunately not helpful. Both reviewers have suggested that you stay closer to your findings when you make recommendations based on your study.

***Authors’ response: Thank you for the clarification. We have changed the language to better emphasize our study findings. Lines 316-319***

Discussion
The authors should discuss their results before relating to others' work. See, for example, lines 215 – 216; what does the willingness of four of five respondents mean in the context of this study?

***Authors’ response: We have rearranged the paragraphs in the discussion section to first discuss the results of this study before relating to others’ work, from lines 235-275***

Reviewer 2:

1. The added sentence is too general and thus does not adequately address the reviewer's comment.

***Authors’ response: Thank you for the clarification. We have added the global, regional and national HIV prevalence and specific key population HIV prevalence in the first paragraph of the introduction to qualify the HIV successes and challenges. Lines 51-70***

2. The response is ok, but you must now add this as a specific study limitation.

***Authors’ response: Thank you for the reminder. We have included information not collected regarding MSM sex work as a study limitation. Lines 304-306***

3. "We have also included the specific HIV preventive methods in the text."  Please specifically indicate where these are these in the text.

***Authors’ response: This information did not appear in the initial manuscript. However, in the revised manuscript we had included the specific biomedical and behavioral HIV preventive methods in lines 142-147***

4. Please indicate that you have incorporated this explanation into the manuscript text (not only in response to the reviewer) and indicate the line number where it appears in the revised manuscript.

***Authors’ response: Please find the following sample size calculation information under METHODS -SAMPLE SIZE lines 103-107***

***A sample size of 250 for the SiVET was calculated based on being able to estimate a one-year retention rate of 80% with a precision of ±5%. The target was to enrol 200 MSM and 50 FSW. The choice to enrol 20% FSW was deliberate because HIV incidence is higher among Kenyan MSM than Kenyan FSW; we also wished to avoid stigma and any hard feelings among our partners at SWOP-Kenya and agreed that enrolling some women would be appropriate.***

7. The reference mentioned as "expanded the discussion citing" is in fact nowhere in the reference list. Also, simply adding 1 reference does not adequately address the reviewer's comment.

***Authors’ response: Thank you for the comment. Reported reasons for not willing to participate have been added in the discussion section of the revised manuscript and the related citations have been included in the reference list. For ease of access please find this information in lines (273-280) and reference list numbers (25, 26, 27, 4, 9 & 12)***

Finally, you have included several comments that you added to your track changed version, however you have not in fact made these changes; it appears that a number of newly added references are missing. Please carefully revise and proofread before resubmission, and make sure to actually add the new references as citations in the text, and to the reference list!

***Authors’ response: Thank you for the comments regarding track changed version. We have reviewed the paper again, and there are corrections and clarifications throughout that we hope make it clearer. The reference list has been reviewed and can confirm the additional citations have been included.***

Line 277 "insert to include refs 11& 12" ??

***Authors’ response: These added citations appear in lines (288& 291) of the revised manuscript and are included in the reference list numbers 14 &15.***

Line 320 - 321  You left in (REF) and (REF). You must actually include the reference number here; and the newly added references must be indicated in the Reference list at the end. There are no apparent changes however to the reference list, despite the claim that a few new references have been added.

***Authors’ response: The lines mentioned 320-321 appear in a paragraph with information related to reported motivators for participation now lines 263-272 in the revised manuscript. The citations reflecting this information is references numbers 19,22,23,16 &, 24.***

Line 328:"insert ref number 3" ??

***Authors’ response: The line 328 appears in a paragraph with information related to reasons for not willing to participate in future trial. This information now appears in lines 273-280 and the references to the information are numbers 4,9,12, 25, 26 & 27***

Please submit your revised manuscript by Aug 06 2020 11:59PM. If you will need more time than this to complete your revisions, please reply to this message or contact the journal office at plosone@plos.org. When you're ready to submit your revision, log on to https://www.editorialmanager.com/pone/ and select the 'Submissions Needing Revision' folder to locate your manuscript file.

Please include the following items when submitting your revised manuscript:

- A rebuttal letter that responds to each point raised by the academic editor and reviewer(s). You should upload this letter as a separate file labeled 'Response to Reviewers'.
- A marked-up copy of your manuscript that highlights changes made to the original version. You should upload this as a separate file labeled 'Revised Manuscript with Track Changes'.
- An unmarked version of your revised paper without tracked changes. You should upload this as a separate file labeled 'Manuscript'.

***Authors’ response: We have reviewed the above and included the above items in our submission.***

If you would like to make changes to your financial disclosure, please include your updated statement in your cover letter. Guidelines for resubmitting your figure files are available below the reviewer comments at the end of this letter.

***Authors’ response: We have reviewed and made changes in the financial disclosure. The updated statement is included in the cover letter.***

***We have reviewed the guidelines of the figure file and confirm we meet these requirements.***

If applicable, we recommend that you deposit your laboratory protocols in protocols.io to enhance the reproducibility of your results. Protocols.io assigns your protocol its own identifier (DOI) so that it can be cited independently in the future. For instructions see: <http://journals.plos.org/plosone/s/submission-guidelines#loc-laboratory-protocols>

***Authors’ response: We did review the above and confirm we meet these requirements. Our*** URL <https://doi.org/10.3886/E119622V1>

***We have also reviewed and expanded the acknowledgment section to include persons/teams who offered their assistance lines 321-325***

***We have also reviewed and included Supporting information S1 Fig.tif Fig 1 in lines 414***
